# Airing Chambers

**Published:** 1876-01

**Authors:** 


AIRING CHAMBERS.

This may be safely done in winter time when the day is clear, at any hour between sunrise and sunset, but on cloudy and damp days it is better to kindle a fire and thus create a draft up the chimney. A bed should always be made several hours before sundown, before it has had time to gather the damps of the evening.

It will refresh us greatly if on waking up of a winter's night, we get out of bed, throw all the clothing to the foot and the next instant throw it back; this drives all the confined air away from the bedding without allowing it to get very cold; in addition the hands should be passed over the skin of the whole body two or three times; this operation is accompanied with a degree of refreshment and a feeling of purity on entering the bed again which more than pays for the trouble, and it is often a great sleep promoter, enabling a person to fall into a sound slumber in a few minutes after having been tossing restlessly for hours.

Shut your mouth in going from a cold to a hot atmosphere, as well as the reverse; this simple operation brings the temperature of either a cold or hot air to the natural standard before it reaches the lungs, by making it take the circuit of the head; whereas if the mouth is kept open it dashes down upon the lungs like a shock. Whether asleep or awake we should accustom ourselves to keep the mouth shut; the advantage in our sleeping hours is that we do not snore, we don't have the nightmare, flies, bugs and spiders don't crawl down the throat, and we don't tell tales in our dreams; the benefits in the day time are that it induces a more healthful, deep, full and free action of the lungs, prevents innumerable chills and colds, and saves many a domestic sorrow.

TEMPERATURE OF ROOMS.

Ordinarily we are comfortable in church, if at the height of five feet from the floor, in the centre of the building, Fahrenheit's thermometer stands at sixty-five degrees. But in this respect no one man should be a guide for another. Some require more heat than others, but there is one rule of universal application—a rule which admits of no exceptions, the world over, each person should notice what temperature keeps him comfortably warm, and thus be a rule to himself. It is said of the Duke of Wellington that during the latter years of his life he became so frigid, that in order to be comfortably warm his room was kept at such a temperature in winter that it was in a measure impossible for any visitor to remain but a very few minutes.

But when a man has taken a cold, or is becoming bilious; or if he stays indoors several days, he requires more and more heat, and if under such circumstances he would eat positively nothing for a day or two and keep on piling up the wood so as to keep up a continual slight perspiration, the cold would be cut short off, or the biliousness would disappear in twenty-four hours; in fact, very many of our aches and pains and ailments would disappear with an amazing promptness, if we could persuade ourselves, when they are first noticed, to only cease eating, keep warm, keep quiet, and drink abundantly of any hot liquid; but the great misfortune is that nine persons out of ten prefer to take some kind of medicine however nauseous; they feel as if they could not spare the time to be sick, and would rather swallow a quart of the most disgusting compound, if it only promises to cure them up "right away," with the result always, that they are not cured right away; but after dosing themselves for days and weeks with whatever Tom, Dick or Harry chooses to advise, they find themselves compelled at last to consult a physician when the time has passed for warmth and quiet to have any curative effect.

### A GREAT MISTAKE.

Many persons precipitate themselves into the grave by attempting to bravado an ailment; to be up and about in defiance of it. If anything at all is the matter with a man which is really disquieting, he should at least have as much sense as a pig, and go and lie down; pigs are not such fools as to move about in pain; it is a great deal better to lie down and

### GRUNT.

Nature, so beautifully appropriate and economical in all her arrangements, makes a double use of the expression of pain or suffering both in men and animals.—a sigh, a moan, a tear, always attract the attention and excite the sympathies of the well and also afford a grateful relief to the sufferer. Those griefs craze the brain which do not vent themselves in weeping. The tearless eye under trouble, breaks the heart. The parent who whips or scolds his child because he cries under suffering or trouble, is a brute or an ignoramus. Neither mirth nor mourning ought to be restrained of their natural expression. Laughter increases the gladness and sighs relieve the sorrowing heart. A very striking exhibition of this idea has frequently come under the observation of the most careless. There is a class of men and women of feeble intellect though they be, who are always complaining; they can entertain you by the hour with the details of their bodily sufferings; according to their own story, they are never free from some ache, or pain, or malady, and you wonder after listening to their interminable narrations that they had not long ago died from the effects of such sufferings; and yet they do not die; they live on, and on, and on, to vex successive generations with their dismal histories; if you sit down to the table with them, you will find that they can eat as much and as fast as the most robust, and have very nice perceptions of the good things of this life, eatable and drinkable. We may, then, reasonably infer that habitual grunting is a life-preserver, and the Frenchman was not very far wrong after all, who advocated the theory several years ago that while it was indisputable that laughing made the body fat, and that good natured children, the lively, merry ones, were healthy, that it was equally certain that crying children were not less so; in other words, that crying did a child good, it promoted a more vigorous circulation of the blood, and helped to develop the lungs.

### GETTING CHILLY.

The reader has no doubt observed many times that if in very severe winter weather he remains in the house several days, the body gets very chilly; while you are warming the feet and hands before the fire, the cold chills run down the back; or if you go even from the fire to the window to look upon the snow, disagreeable creeps run all over the body; and whether in these, or under any other circumstances, persons have an unpleasant chilliness, it is the result of a sluggish circulation and an imperfect digestion—so little life-giving air is breathed, and so little exercise is taken that the nutriment is not drawn from the food eaten, the blood grows poor, and lifeless, and cold; loses its heating power, and the body begins to freeze and die. But let a few hours be spent in the cool, out-door air in some exhilerating employment or pastime, and there is an entire change in the whole physical and mental condition; the fire of life kindles in the eye, smiles light up the face, and the man is himself again.

